# Cardiac Safety of Kinase Inhibitors – Improving Understanding and Prediction of Liabilities in Drug Discovery Using Human Stem Cell-Derived Models

**DOI:** 10.3389/fcvm.2021.639824

**Published:** 2021-06-16

**Authors:** Ricarda Ziegler, Fabian Häusermann, Stephan Kirchner, Liudmila Polonchuk

**Affiliations:** Pharmaceutical Sciences, Pharma Research and Early Development, Roche Innovation Center Basel, F. Hoffmann-La Roche Ltd., Basel, Switzerland

**Keywords:** kinase inhibitors, cardiotoxicity, iPS-derived cardiomyocytes, apoptosis, beating rate, cell impedance, CardioExcyte®96

## Abstract

Many small molecule kinase inhibitors (SMKIs) used to fight cancer have been associated with cardiotoxicity in the clinic. Therefore, preventing their failure in clinical development is a priority for preclinical discovery. Our study focused on the integration and concurrent measurement of ATP, apoptosis dynamics and functional cardiac indexes in human stem cell-derived cardiomyocytes (hSC-CMs) to provide further insights into molecular determinants of compromised cardiac function. Ten out of the fourteen tested SMKIs resulted in a biologically relevant decrease in either beating rate or base impedance (cell number index), illustrating cardiotoxicity as one of the major safety liabilities of SMKIs, in particular of those involved in the PI3K–AKT pathway. Pearson's correlation analysis indicated a good correlation between the different read-outs of functional importance. Therefore, measurement of ATP concentrations and apoptosis *in vitro* could provide important insight into mechanisms of cardiotoxicity. Detailed investigation of the cellular signals facilitated multi-parameter evaluation allowing integrative assessment of cardiomyocyte behavior. The resulting correlation can be used as a tool to highlight changes in cardiac function and potentially to categorize drugs based on their mechanisms of action.

## Introduction

Over the last decade, small molecule kinase inhibitors (SMKIs) development has gained more importance due to the mechanistic role of kinases in various diseases and their ability to control basic functions in normal cells (1). Kinases are involved in a number of essential cellular functions such as mitosis, apoptosis and cell signaling (2). Approximately a dozen were marketed for applications in oncology and several for various other indications (3, 4). Because most SMKIs target the structurally and functionally conserved ATP binding pocket, their inhibitory effect has been associated with toxicities that result from exaggerated pharmacological effect or unselective off-target kinase inhibition. These off-target safety issues remain a major cause for failure in drug development. The unintended inhibition of off-target kinases can lead to undesirable toxicities, which may discourage further drug development (5).

Many small molecule kinase inhibitors have been associated with cardiotoxicity in clinical development and preclinical discovery (6, 7). The cardio-toxicological potential of SMKIs may arise from direct or indirect kinase inhibition, or from the off-target effects. Over 2,000 other nucleotide-dependent enzymes besides kinases exist, including polymerases, chaperones, motor proteins, reductases and methyltransferases, which also have potential binding sites for SMKIs (8). An extensive range of kinase-regulated pathways are involved in essential functions of the heart and could trigger cardiotoxicity when inhibited. For example, cardiovascular energy homeostasis is predominantly regulated by protein kinases that maintains a balance of ongoing energy generation and use essential to avoiding congestive heart failure (CHF) (9). Another important pathway is calcium homeostasis, where calcium/calmodulin-dependent protein kinase II is a key player (10). Any disturbance to calcium regulation can lead to altered cardiac conduction and cardiac hypertrophy (11). The most critical interference in a pathway is with the (PI3K)–AKT pathway which regulates cardiomyocyte survival (12). However, inhibition of specific PI3K–AKT pathway compounds is a common strategy in cancer treatment (13). Moreover, a kinase inhibitor-mediated toxicity mechanism can ablate or suppress the proliferation of protective cell compartment important for heart repair (14). In cancer patients following treatment with SMKIs, adverse cardiac events such as QT prolongation, hypertension, reduced left ventricular ejection fraction (LVEF), congestive heart failure (CHF), acute coronary syndromes (ACS), and myocardial infarction (MI) were often observed (15, 16). In the past, cardiotoxicity could be only addressed with *in vivo* models, missing many earlier toxicological cues leading to cardiovascular safety pharmacology liabilities, which were then identified in late-stage clinical trials or even after product launch (17). With the development of human stem cell-derived cardiomyocytes (hSC-CMS), it became possible to study cardiac function by monitoring synchronous cardiac cell beating and behavior *in vitro* (17). Cellular assays with hSC-CM are proved to be predictive for main functional cardiotoxicity hazards such as arrhythmia, chronotropy, inotropy, and kinase inhibitor induced functional cardiotoxicity, underscoring the importance of cardiotoxicity screening during drug development (18–20). Recently, novel cellular electrophysiology technology enabling simultaneous measurements of cellular impedance and extracellular field potential (EFP) has been implemented to monitor cardiac functional indices *in vitro* (21). CardioExcyte® 96 (Nanion Technologies GmbH, Germany) is a screening platform combining impedance and extracellular field potential (EFP) recordings, which allows label-free simultaneous assessment of electrical activity, beating and electromechanical coupling. Combining standard monitoring cardiomyocyte beating and health with live-cell kinetic imaging and the ATP measurements helps us to gain a valuable, more mechanistic understanding of the compound effect during early stage cardiotoxicity investigations. In this study a selected set of reference SMKIs included low and high selectivity inhibitors targeting various classes of PI3K, GSK-3 and ROCK kinases.

Our study focused on the integration and concurrent measurement of cardiomyocyte beating, health, ATP and apoptosis dynamics in hSC-CMs to gain insights into molecular determinants of compromised cardiac function. The results of this work should help to define optimum end- and time-points for follow up experiments and to design a strategy for compound profiling in early drug development.

## Materials and Methods

### Chemicals and Compounds

Compounds were obtained from commercial suppliers (Table 1). Stock solutions (1000x) were prepared in DMSO (cat# D5879, Sigma-Aldrich, St. Louis, Missouri) and the final DMSO concentration was 0.1% v/v. hSC-CM were treated with ascending concentrations of compounds with the highest test concentrations set based on individual compound solubility. 0.1% v/v DMSO was used as a vehicle control.

**Table 1:** Test compounds.

**Compound**	**Supplier**	**CAS-Number**	**LOT Number**
NVP-BEZ235	Cayman Chemical; Ann Arbor, Michigan	915019-65-7	048902-2
LY 294002	Toronto Research Chemicals; North York, Canada	154447-36-6	1-XGI-65-1
GDC-0941	Toronto Research Chemicals; North York, Canada	957054-30-7	1-XGI-79-1
HS-173	Toronto Research Chemicals; North York, Canada	1276110-06-5	2-TIM-79-1
TGX-221	Toronto Research Chemicals; North York, Canada	663619-89-4	1-NAZ-38-1
CZC24832	Fluorochem; Glossop, United Kingdom	1159824-67-5	FCB041717
CAL-101	Cayman Chemical; Ann Arbor, Michigan	870281-82-6	0469747-22
RKI-1447	Lucerna-Chem AG; Luzern, Switzerland	1342278-01-6	10020
GSK-429286	Sigma-Aldrich; St. Louis, Missouri	864082-47-3	012M4614V
Y-27632 (hydrochloride)	Cayman Chemical; Ann Arbor, Michigan	129830-38-2	0499111-18
Fasudil	Alfa Aesar; Ward Hill, Massachusetts	103745-39-7	F23X003
CHIR99021	Sigma-Aldrich; St. Louis, Missouri	252917-06-9	017M4717V
SB 216763	Abcam; Cambridge, United Kingdom	280744-09-4	APN08058-2-3
TWS119	Cayman Chemical; Ann Arbor, Michigan	601514-19-6	0475327-29

### Cell Culture

Cor.4U® Cardiomyocytes (Ncardia, Netherlands) were cultured in 96-well plates at density of 25,000 – 30,000 cells per well. Cells were maintained at 5% CO_2_ and 37 °C for 4 days prior to experiments. The cell culture medium (Cor4U® Complete Culture Medium, cat# Ax-M-HC250E, Axiogenesis AG, Cologne, Germany) was changed every 24 h and was replaced by serum-free BMCC medium (cat# Ax-M-BMCC259, Axiogenesis AG, Cologne, Germany) 12 h prior to drug addition.

### CardioExcyte® 96 Experiments

Functional monitoring of cardiomyocytes was done using CardioExcyte® 96 (Nanion technologies GmbH, Germany). Extracellular field potential (EFP) and impedance signals were recorded from hSC-CMs that formed monolayers in the wells of the NSP-96 plate. Beating Rate (BR, per minute) was calculated as the reciprocal of the inter-spike interval (ISI) in EFP signal. Impedance signals from spontaneously beating hSC-CMs were recorded with a sampling rate of 1 ms (1 kHz), and EFP data were collected at 0.1ms (10 kHz). Data acquisition was controlled by CardioExcyte® 96 Control software as previously described (22). The functional parameters recorded in each concentration of test items were compared to the base line values before drug addition (taken as 100%) to define fractional change.

### Apoptosis Assay

Determination of apoptosis kinetics was performed with the CellEvent^TM^ Caspase-3/7 Green Detection reagent (cat#1825104, Molecular probes®/Invitrogen™) in BMCC medium over 24 h. The plates were incubated 2 h before compound treatment in the IncuCyte ZOOM (ESSEN BioScience, Ann Arbor, Michigan) to set a baseline before the 24 h compound treatment. Staurosporine (1 μM, cat# 058K4041, Sigma-Aldrich, USA) was used as positive control. Imaging was done using 10x microscope objective of the IncuCyteZOOM with four images per well-taken every 2 h in both phase-contrast and green fluorescence. The fluorescent nuclear signal of the apoptotic cells was separated from background with the integrated object counting algorithm, adjusted to cardiomyocytes, and the calculated total green object area per well (μm^2^/well) plotted against time. Data were normalized to those of the time-matched vehicle control. Data were representative of at least three replicates per concentration.

### ATP Assay

The CellTiter-Glo Luminescent Cell Viability Assay (cat# G7572, Promega, USA) was performed for quantification of the cellular ATP concentration according to manufacturer's instructions. Plates were shaken for 2 min in the dark, incubated at ambient temperature for 10 min before measuring luminescence (Integration time 0.1 seconds per well) with Luminescence Counter (Synergy H1, BioTek Instruments, Inc., Winooski, Vermont). The measured ATP concentration values were normalized to the time-matched DMSO controls. Data are representative of at least three replicates per concentration.

### Data Analysis

All data were presented as mean ± SEM for each test item concentration and controls. Graphical presentation and statistical analysis of the data by one-way ANOVA with Dunnett multiple comparison or by paired *t*-test were done using Microsoft Excel (USA) and GraphPad Prism v8.4.2 (USA).

## Results

### Integrative Study Design

**Figure 1:**
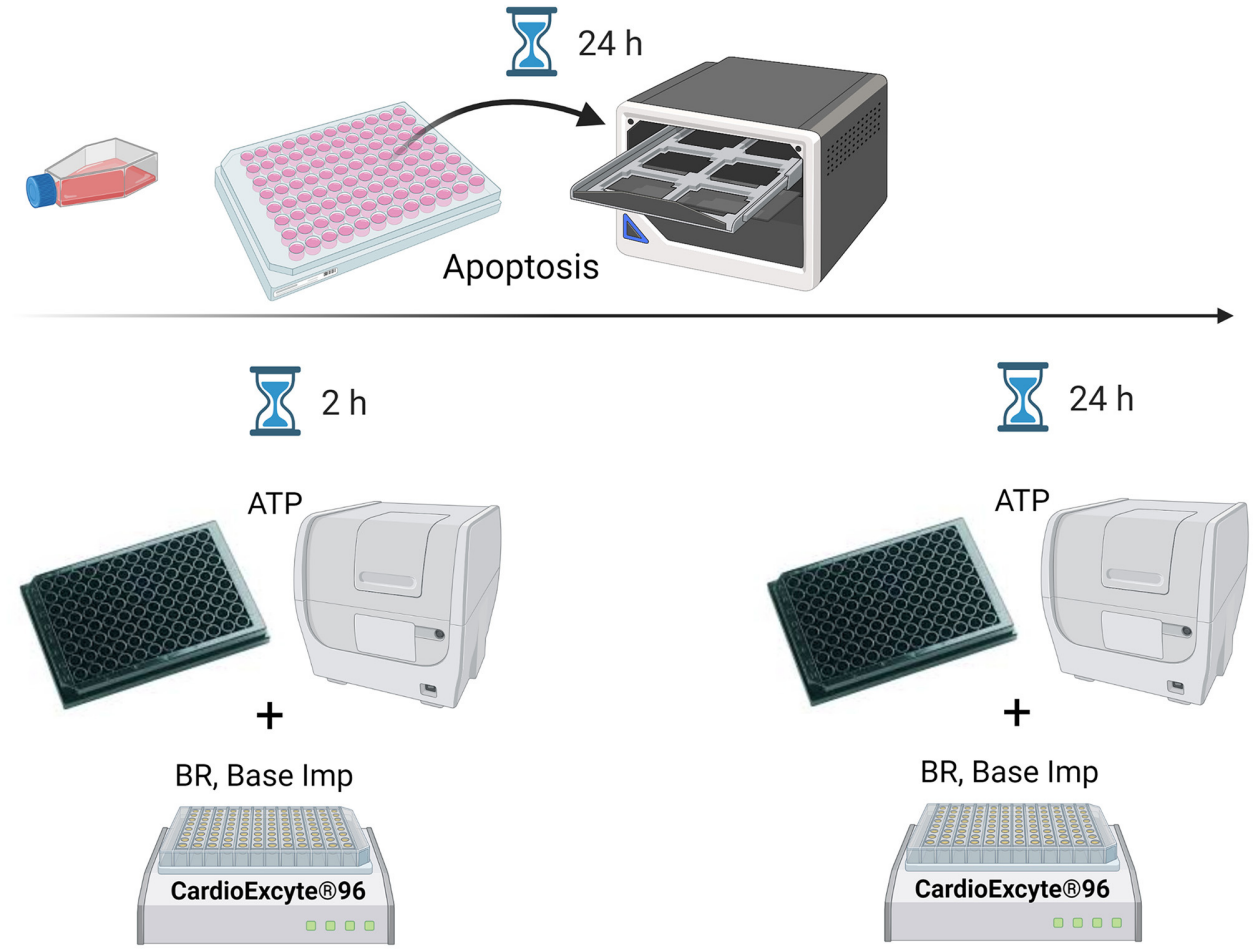
Scheme of assay-specific measurement time points. The ATP and CE96 measurements were conducted 2 and 24 h after the compound treatment whereas apoptosis was continuously monitored over 24 h. BR, beating rate; Base Imp, base impedance.

In the present study, we used CardioExcyte® 96 for multi-parameter profiling of endogenous responses to 14 SMKIs in hSC-CMs. Cor4U® hSC-derived cardiomyocytes were treated with pan and specific PI3K, ROCK and GSK-3 kinase inhibitors to evaluate short-and long-term effects on impedance and EFP signals after 2 and 24 h, respectively. Complementary to the functional effects on the cellular beating and dynamics in culture, continuous measurement of apoptosis during the compound incubation was performed over 24 h with the Caspase 3/7 assay. Determination of the cells' ATP content was done after 2 and 24 h as visualized in Figure 1.

### Effects of Kinase Inhibitors on hSC-CMs Characteristics

#### Apoptosis

**Figure 2:**
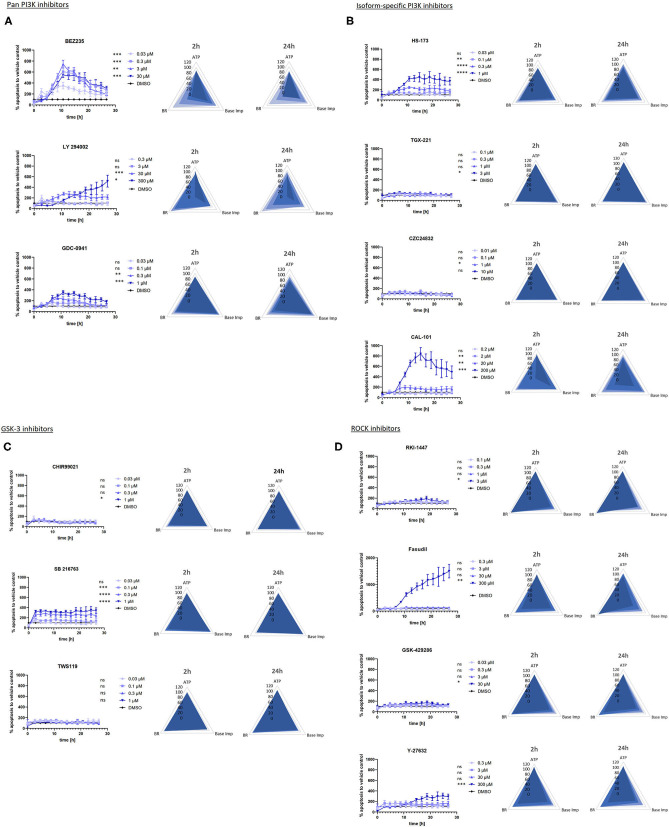
Graphs of apoptosis kinetics and radar plots for beating rate, base impedance and ATP measured in the hSC-CMs treated with SMKI. **(A–D)** show the results for the PI3K-pan, PI3K isoform-specific, GSK-3 and ROCK kinase inhibitors, respectively. The data represent the average of 3–5 repeats for each time point. The apoptosis graphs show mean +/- SEM of kinetic data sampled every 2 h after the addition of compounds. The fractional change from baseline determined for each concentration is compared to that of vehicle control (DMSO). The significance is indicated by asterisk (or ns for p>0.1) next to the specific concentration in the graph legends: *p < 0.1, **p < 0.01, ***p < 0.001, ****p < 0.0001. Two-dimensional radar charts present multivariate drug-induced functional profiles in hSC-CMs. They display fractional change from baseline in beating rate, base impedance and ATP content across ascending concentrations of drugs after the short- (2 h) and long-term (24 h) treatment.

Incucyte® live-cell imaging and analysis enabled us to continuously monitor and evaluate apoptotic effects in the hSC-derived myocytes treated with a diverse panel of kinase inhibitors over 24 h. A concentration-dependent increase in apoptosis was detected by caspase-3/7 assay for 10 out of 14 compounds with 5 of them belonging to the group of PI3K inhibitors. An overview of the individual compound results is presented in Figure 2, which consists of 4 panels built according to the kinase class and specificity of the inhibitors. Panels A, B, C and D show the results for the PI3K-pan, PI3K isoform-specific, GSK-3 and ROCK kinase inhibitors, respectively. Individual data used to generate graphs are provided in Data File 1. 1μM used as a positive control induced a strong caspase 3/7 signal that could be monitored over 24 h in hSC-CMs (Supplementary Figure 1).

The activation of caspase-3/7 represents a key event and a reliable marker of apoptosis. Activated caspase-3/7 cleaves the caspase-3/7 Green Reagent and releases a DNA-binding dye to identify apoptotic cells by the appearance of fluorescently-labeled nuclei. The accumulation of apoptotic cells was plotted as a function of time to analyze the kinetics of apoptosis in hSC-CMs incubated with the set of kinase inhibitors. This analysis revealed apoptotic profiles specific for the targeted kinase-class.

The pan-PI3K inhibitors NVP-BEZ235, LY 294002, GDC-0941 (Figure 2A), PI3K p110α inhibitor HS-173 and PI3K p110δ inhibitor CAL-101(Figure 2B) induced apoptosis in hSC-CMs with a fast onset reaching the maximum number of apoptotic cells in the first 12 h, whereas the PI3K p110β and PI3K p110γ-specific compounds (TGX-221 and CZC24832) had no effect (Figure 2B). The concentration-dependent caspase-3/7 activation exhibited similar kinetic profiles across all PI3K inhibitors except for the top concentration of pan-PI3K inhibitor LY 294002 which induced a delayed apoptotic response in hSC-CMs.

GSK-3α kinase inhibitor SB 216763 quickly induced a concentration-dependent caspase−3/7 activation in hSC-CMs (Figure 2C), which reached a plateau within the first hour and was maintained for the remaining time. The other two GSK-3 kinase inhibitors CHIR99021 (GSK-3α/β) and TWS119 (GSK-3 β) did not have any functionally-relevant effect up to the highest concentrations tested.

Treatment hSC-CMs with ROCK inhibitors Fasudil and Y-27632 (Figure 2D) triggered a different apoptotic behavior characterized by a slow initial phase followed by a notable raise in the caspase-3/7 activity after 12–18 h at the highest test concentrations only. In general, hSC-CMs seemed to be more resistant to the apoptosis induced by the ROCK inhibitors, as exemplified by marginal effects observed for RKI-1447 and GSK-429286 at the highest test concentrations only.

#### Beating Rate and Base Impedance

In this study, monitoring of the hSC-CMs functional activity was done using the CardioExcyte® 96 platform from Nanion technologies GmbH, Germany, which combines impedance and EFP recordings to study pharmacological effects on cardiomyocyte electrophysiology with a sufficient throughput for testing of multiple parallel compounds. Base impedance (or the absolute impedance recorded from each well) was a key parameter for estimation of cell viability. Decrease of this parameter is considered as a surrogate for reduced viability of hSC-CMs. Base impedance and beating rate were recorded in combination to characterize impact of the kinase inhibitors on the cardiac function after 2 and 24 h. Effects of individual compounds are summarized in the corresponding radar plots shown in Figures 2A–D. The functional parameters recorded in each concentration of the test items were normalized to the base line values before drug addition to define fractional change. Individual data used to generate radar plots are presented in Data File 1.

Treatment with two pan-PI3K inhibitors NVP-BEZ235 and LY 294002, the PI3K p110δ inhibitor CAL-101 and the PI3K p110α inhibitor HS-173 reduced both base impedance and beating rate of hSC-CMs by more than 20% (Figures 2A,B), whereas the pan-PI3K inhibitor GDC-0941 only decreased the spontaneous beating rate in a concentration-and time-dependent manner. The PI3K p110β and PI3K p110γ-specific compounds (TGX-221 and CZC24832) did not have any effect on the hSC-CMs function.

The GSK-3 inhibitors CHIR99021, SB 216763 and TWS119 (Figure 2C) did not produce functionally relevant changes neither in base impedance nor in beating rate of hSC-CMs.

Incubation of hSC-CMs with ROCK inhibitors RKI-1447, Fasudil, Y-27632 and GSK-429286 (Figure 2D) resulted in a concentration-and time-dependent decrease in base impedance. Additionally, the Y-27632 treatment also reduced cardiomyocyte beating at both 2 h and 24 h time points, while Fasudil inhibited the beating rate (by ~30%) only at the highest test concentration after 2 h of incubation.

#### ATP Level

Changes in ATP concentrations provoked by treatment of hSC-CMs with the kinase inhibitors are also included in radar plots in Figures 2A–D. The measurements were conducted in satellite plates after 2 and 24 h of incubation concomitantly with the readouts for the base impedance and beating rate.

The ATP level in hSC-CMs treated with pan-PI3K inhibitors NVP-BEZ235, LY 294002, GDC-0941, PI3K p110α inhibitor HS-173 and PI3K p110δ inhibitor CAL-101 was decreased by ~20–25 % only after 24 h of incubation (Figures 2A,B). And the PI3K p110β and PI3K p110γ-specific compounds (TGX-221 and CZC24832) did not have any effect on the ATP level in hSC-CMs up to the highest test concentrations.

Similar to the absence of their effect on functional parameters, the GSK-3 inhibitors CHIR99021, SB 216763 and TWS119 did not impact the ATP level in hSC-CMs (Figure 2C).

Among ROCK inhibitors RKI-1447, Fasudil, Y-27632 and GSK-429286, only exposure to the Fasudil top concentration produced a slight (9%) reduction in the ATP content (Figure 2D).

### Correlation Between the hSC-CM Characteristics

**Figure 3:**
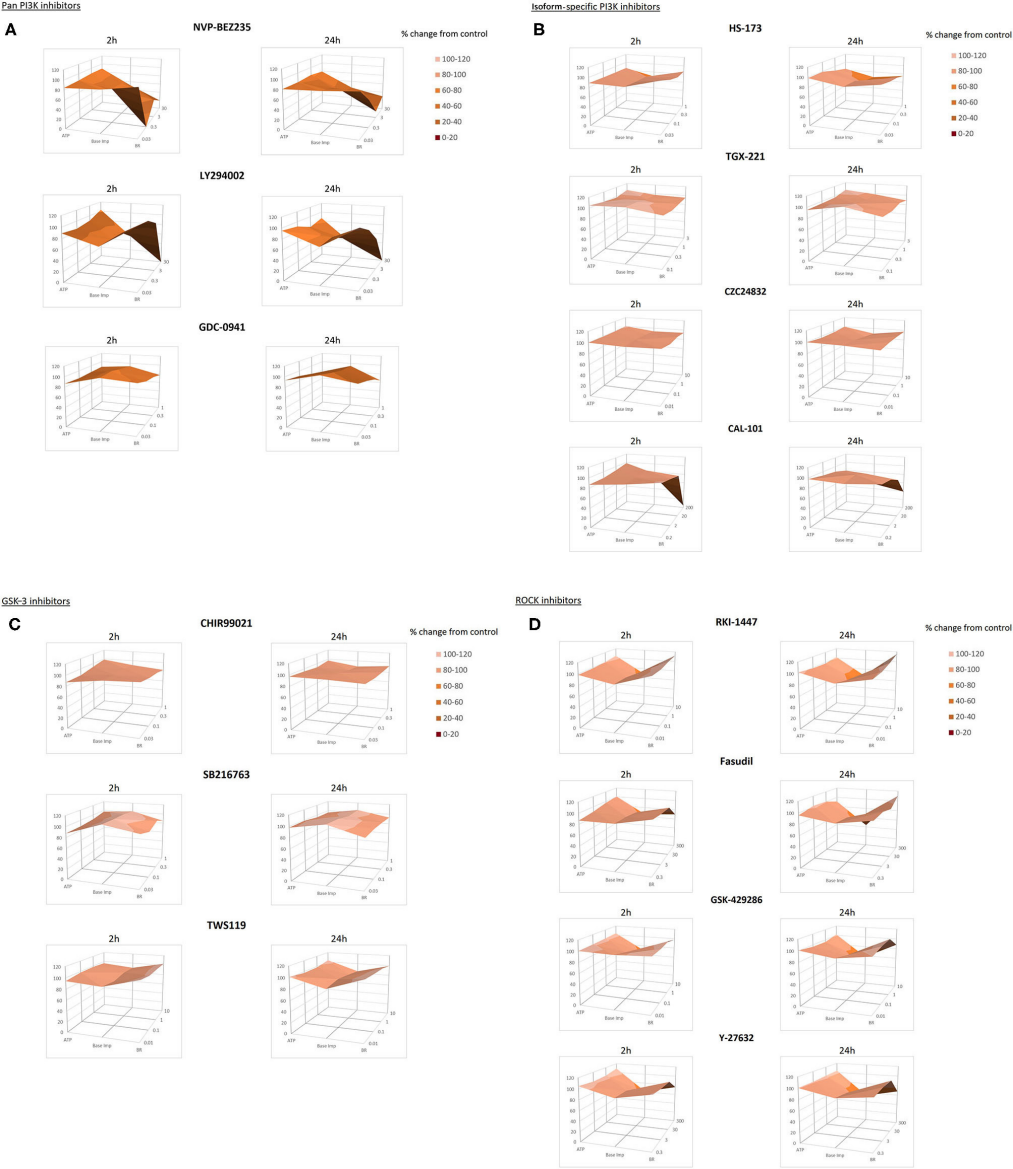
Surface plots for beating rate, base impedance and ATP measured in hSC-CMs after 2 and 24 h of incubation with kinase inhibitors. **(A–D)** show the results for the PI3K-pan, PI3K isoform-specific, GSK-3 and ROCK kinase inhibitors, respectively. Surface plots intend to show compound-specific relationships between individual functional parameters in hSC-CMs: beating rate, base impedance and ATP content. The plots are constructed using the average of 3–5 repeats for each parameter. Similar colors indicate the areas that are in the same range of values specified in the legend.

Changes in base impedance, beating rate and ATP described above were plotted on a three-dimensional surface to visualize potential interactions and compare short-(2 h) and long-term (24 h) effects of the kinase inhibitors on all three parameters. The resulting surface plots are shown in Figure 3 sorted according to the kinase class and specificity of the inhibitors: panels A, B, C and D show the results for the PI3K-pan, PI3K isoform-specific, GSK-3 and ROCK kinase inhibitors, respectively. The colors represent values within the same range in 20%-step intervals. The three-dimensional surface is constructed using the average values of parameter changes. In the absence of the effect the surface is perfectly flat, while morphology, smoothness, and topological measures of the surface allow us to visualize the parameter changes. When looking for trends and comparing compound-, target- or time-specific changes in the sets of data, this visualization was especially useful.

Comparison of the surfaces across the set of kinase inhibitors highlighted pronounced effects of the pan-PI3K inhibitors NVP-BEZ235 and LY 294002 and the PI3K p110δ inhibitor CAL-101 on the cardiomyocyte function reflected by the abrupt shape and shading change (Figures 3A,B). At the same time the surfaces generated for the pan-PI3K inhibitor GDC-0941, the PI3K p110β inhibitor TGX-221 and the PI3K p110γ-specific inhibitor CZC24832 exhibited only a minor topographical remodeling reflecting physiologically non-relevant change below 20% in the hSC-CMs parameters.

The almost flat 3D surfaces for the GSK-3 inhibitors CHIR99021, SB 216763 and TWS119 (Figure 3C) demonstrated minimal functional effect of these compounds in hSC-CMs. And the specific surface deformation for ROCK inhibitors RKI-1447, Fasudil, Y-27632 and GSK-429286 (Figure 3D) spotted the concentration-dependent effects of these compounds on the cardiomyocyte base impedance/viability.

In general, these 3D surface plots were helpful for viewing the relationship among variables, while also assisting in the initial qualitative comparisons between the subsets of data. Subsequently, Pearson's coefficient matrix presented in Table 2 was calculated to quantify the correlation showing the relationship between the parameters measured in the study for 8 compounds that induced apoptosis and notable changes in the hSC-CMs function and the ATP level.

**Table 2:** Pearson's coefficient matrix between sets of parameters in hSC-CMs.

	**2h**						**24h**					
**Compound**	**Caspase: ATP**	**Caspase: Base Imp**	**Caspace: BR**	**BR: ATP**	**BR: Base Imp**	**Base Imp: ATP**	**Caspase: ATP**	**Caspase: Base Imp**	**Caspace: BR**	**BR: ATP**	**BR: Base Imp**	**Base Imp: ATP**
BEZ235	−0.83	0.74	0.93	−0.91	0.93	−0.88	−0.78	−0.51	−0.40	−0.12	0.92	0.18
LY 294002	−0.70	0.80	0.82	−0.97	1.00	−0.96	−0.46	−1.00	−0.99	0.51	0.98	0.44
GDC-0941	−0.39	0.86	0.93	−0.35	0.86	−0.78	−0.93	−0.71	−0.94	1.00	0.91	0.92
HS-173	0.99	0.77	0.99	0.97	0.71	0.85	−0.93	−0.91	−0.98	0.92	0.94	0.98
CAL-101	−0.93	0.67	0.81	−0.94	0.97	−0.84	−0.93	−1.00	−0.99	0.97	0.98	0.90
SB 216763	0.96	−0.96	−0.52	−0.61	0.73	−0.97	−0.82	−0.71	0.42	−0.16	−0.62	0.83
Fasudil	−0.98	0.82	0.78	−0.81	0.99	−0.86	−0.84	−0.95	0.92	−0.75	−0.77	0.73
Y-27632	−0.72	0.71	0.53	−0.88	0.96	−0.85	0.95	−0.94	−0.89	−0.76	0.73	−1.00

*Values are formatted using 2-color scale in the range from−1(min) to 1(max). BR, beating rate; Base Imp, base impedance*.

The results showed a good overall correlation among four measured parameters with 91% of the Pearson's coefficient values being between ± 0.50 and ± 1. And some of these quantitative indexes had direct physiological relevance for the cellular process. For instance, correlation between the caspase-3/7 activation and the ATP level was mainly negative indicating an inverse relationship, which most likely reflected the ATP consumption during apoptosis, while impedance and beating rate demonstrated a strong positive correlation indicating the concomitant reduction in cell viability and beating rate.

Interestingly, a time-dependent switch was observed for some parameter pairs. For example, initial caspase-3/7 activation by the kinase inhibitors correlated positively with base impedance and beating rate for almost all compounds after 2 h of incubation. After 24 h the relationship inverted, and the correlation became mainly negative highlighting decline in function with the advance of apoptosis in the cells. The opposite tendency was observed for the correlation between base impedance and the ATP level, which was negative at the early stage and became positive after the prolonged exposure to the compounds. This dynamic highlighted different mechanisms involved in short-vs. long-term effects.

A few compounds such as SB 216763, Fasudil and sometimes HS-173 did not follow these general trends in the parameter correlation. These exceptions may, on one hand, be due to the minimal changes in parameter values and of no physiological importance. On the other hand, they may reflect unknown compound-specific mechanisms of action.

### Translation of the *in vitro* Results Into the Clinic

Among the test compounds evaluated in our study, three PI3K inhibitors Dactolisib, Pictilisib and Idelalisib have been used for treatment of malignancies in the clinic. According to the information collated in Table 3, the test concentrations used in the *in vitro* cardiomyocyte study covered the range of compound efficacy in patients. The latter was substantially higher than their IC_50_-based biological activity determined in the cancer cell-based systems.

**Table 3:** Compound biological activity and efficacy information.

**Inhibitor**	**Target IC**_****50****_ **(μM)**				**Test conc. (μM)**	**C_**max**_ free (μM)**	**Reference**
**PI3KIs**	**p110α**	**p110β**	**p110δ**	**p110γ**			
NVP-BEZ235 (Dactolisib)	0.004	0.075	0.007	0.005	0.03 **- 0.3 -** 3 **-** 30	**0.02**	IC_50_: (23–25) C_max_: (26)
LY 294002	0.5	0.973	0.57		0.3 - 3 - 30 - 300		(27)
GDC-0941 (Pictilisib)	0.003	0.033	0.003	0.075	0.03 - 0.1 - 0.3 - **1**	**0.1**	IC_50_: (28) C_max_: (29, 30)
HS-173	0.0008				0.03 - 0.1 - 0.3 - 1		IC_50_: (31)
TGX-221		0.005			0.1 - 0.3 - 1 - 3		IC_50_: (32)
CAL-101 (Idelalisib)	0.82	0.056	0.0025	0.089	0.2 - 2 - **20** - 200	**0.7**	IC_50_: (33) C_max_: (34)
CZC24832		1.1		0.027	0.01 - 0.1 - 1 - 10		IC_50_: (35)
**ROCKs**	**ROCK1**	**ROCK2**					
RKI-1447	0.0145	0.0062			0.1 - 0.3 - 1 - 3		IC_50_: (36)
Fasudil	0.465	0.509			0.3 - 3 - 30 - 300		IC_50_: (37, 38)
Y-27632	0.093	0.089			0.3 - 3 - 30 - 300		IC_50_: (39–43)
GSK-429286	0.014	0.063			0.03 - 0.3 - 3 - 30		IC_50_: (44, 45)
**GSK-3s**	**GSK-3α**	**GSK-3β**					
CHIR99021	0.01	0.0067			0.03 - 0.1 - 0.3 - 1		IC_50_: (46)
SB 216763	0.034				0.03 - 0.1 - 0.3 - 1		IC_50_: (47)
TWS119		0.03			0.03 - 0.1 - 0.3 - 1		IC_50_: (48)

*The lowest in vitro test concentration with functionally relevant (>20%) reduction in hSC-CMs indices is indicated by the bold format*.

A 10-fold and higher safety margin between the clinical exposure and the lowest *in vitro* test concentration with functionally relevant (>20%) reduction in hSC-CMs indices (underlined in Table 3) can be estimated for these drugs. Therefore, one could expect cardiac adverse events if the free plasma concentration increases 10-fold above the expected free C_max_ e.g., due to risk factors and/or comorbidities in the clinic.

## Discussion

Not only does predicting drug-induced cardio toxicity remain a pharma-industry challenge, it is also one of the major causes of drug withdrawal during clinical development, accounting for up to 33% of drug failure (49). Because regenerative capacity of the heart ceases dramatically after birth and the sources of primary adult cardiomyocytes are limited, commercially available human stem cell-derived cardiomyocytes hold great promise for the study of cardiac biology, pharmacology and safety in drug development.

To make cardiotoxicity testing more biologically relevant, the FDA-sponsored Cardiac Safety Research Consortium and the not-for-profit Health and Environmental Sciences Institute have recommended, as a part of the Comprehensive *in vitro* Proarrhythmia Assay (CiPA) initiative, including hSC-CMs into the nonclinical drug cardiotoxicity assessment with the goal of using them as an integrating model system bridging single receptor-based *in vitro* results and clinical data (50).

While the use of hSC-CMs for prediction of drug-induced arrhythmias has been recently evaluated (51), their application for cardio-oncology is still unexplored. This study attempted to look into utility of hSC-CMs at the pre-clinical stage to predict cardiac adverse effects of SMKIs used for targeted anti-cancer therapy in the clinic.

A set of reference drugs inhibiting individual kinases from the main three classes (PI3Ks, GSK-3s and ROCKs) critical for cardiac function was selected to evaluate the action of their inhibition on hSC-CMs function. In addition to the direct functional readouts of cell viability and beating rate on CardioExcyte® 96, measurements of apoptosis kinetics and ATP were integrated into the experiments enabling mechanistic insight.

An early event in apoptosis is activation of caspases, of which caspase-3 is the most critical apoptotic protease playing an important role in apoptosis of myocardial cells with functional importance (52). Therefore, we monitored the induction of caspase-3/7 to investigate the involvement of SMKIs in apoptosis in hSC-CMs. The kinase-specific caspase-3/7 activation profiles were observed using live-cell imaging analysis for the first time in these cells. They demonstrated the importance of the kinase signaling pathway and selectivity of SMKIs for the apoptosis development in hSC-CMs. Targeting of multiple kinases with the pan-PI3K inhibitors to achieve maximal suppression of activity of that pathway may be a favorable approach in a number of cancers. However, this pathway obviously is also crucial for cardiomyocyte homeostasis and survival, and its inhibition quickly triggers apoptosis resulting in reduced viability and beating of the cardiomyocytes observed for the pan-PI3K inhibitors in this study. Improving the isoform selectivity may help to ameliorate the cardiotoxicity profile of drug candidates as observed in hSC-CMs incubated with the PI3K-β and γ-specific inhibitors. This observation supports the published evidence of cardioprotective effects of the PI3K-α inhibition in heart failure (53) and doxorubicin-induced cardiotoxicity (54). However, this does not apply to all isoforms. The cardiotoxic effects observed in hSC-CMs incubated with HS-173 clearly illustrates a decisive role the PI3K-α signaling inhibition plays in the process of cell death in the heart. In line with our finding, previous studies demonstrated that the use of PI3K-α inhibitors may directly or indirectly compromise cardiac function (55). The PI3K-α pathway inhibition promoted heart atrophy, biventricular remodeling, and increased susceptibility to doxorubicin toxicity in mice (56). Rescue treatment using p38 MAPK inhibitors showed potential as a therapeutic strategy in reversing this chemotherapy-induced cardiotoxicity. Early signal detection in hSC-CMs would be particularly important for risk mitigation and disease modeling since many cardioprotective effects of the PI3K signaling pathways only emerge in heart failure (53, 57, 58) and patients with pre-existing cardiac disease or risk factors, such as hypertension or metabolic syndrome may be at higher risk when given PI3K-α inhibitors. And early clinical studies may not fully appreciate the cardiovascular effects of these agents in the vulnerable population.

Tissue specificity may be another way to avoid potential cardiotoxicity. For instance, PI3Kδ, which is mainly expressed in hemopoietic cells, may be devoid of possible side effects in cardiomyocytes. Conversely to this expectation, the PI3Kδ inhibitor still induced apoptosis, which correlated with the reduced cardiomyocyte viability and beating rate in our hSC-CMs experiments. However, as CAL-101 triggered cardiotoxicity mainly at higher concentrations, it may also be related to the concentration-dependent decrease in selectivity similar to that of the pan-PI3K inhibitors (see Table 3).

Shortly after the start of treatment with the GSK-3α inhibitor, a pronounced surge in the caspase activation was detected. However, this surge in activation did not translate into the relevant functional changes in hSC-CMs. This caspase activation pattern may reflect the GSK's regulation by AKT that induces pro-apoptotic factors downstream of PI3Ks. The inhibition of PI3K-α and GSK-3 α is cytotoxic in hSC-CMs. Therefore, compounds that interfere with the PI3K–AKT pathway should be carefully examined, because they can cause cardiotoxicity and cardiovascular effects.

Altogether, our findings in hSC-CMs suggest multiple, critical kinase-regulated pathways that, if inhibited, could aggravate cardiotoxicity. Therefore, an early target assessment including the expression profile in various tissues and tumors and target selectivity (e.g., KinomeScan) profiling of the SMKIs against multiple targets should help eliminating late-stage cardiotoxicity findings. And hSC-CMs-based assays would enable further *in vitro* testing of adverse effects induced by different SMKIs in combination with other cancer therapies and comorbidities.

Development of integrative functional assays for kinase profiling by multiplexing apoptosis measurement with functional readouts and the ATP assessment can enable researchers to better predict cardiotoxicity potential and/or elucidate an underlying mechanism. Implementation of the next generation hybrid biosensor equipment (e.g., Agilent xCELLigence RTCA eSight), which combines the strengths of real-time impedance monitoring with specificity of the live cell imaging, would facilitate integration of the information richness and analytic sensitivity. Correlation between the parameters in hSC-CMs can also assist in characterizing the basic physiology and pharmacology of these cells and their utility for cardiac safety application.

The evaluated cellular endpoints exhibited a good, and physiologically meaningful, correlation. For example, beating rate and impedance demonstrated a strong positive correlation that reflected the slowing of the beating rate following the reduction in cell viability.

Furthermore, the ATP increase which accompanied the caspase-3/7 activation at the early stage (2 h) was reversed with the advance of apoptosis. Beating cardiomyocytes consume vast amounts of ATP. The average ATP concentration determined in the hSC-CM under control conditions was around 5 μM. It is utilized to maintain cellular homeostasis including the necessary gradients of ions driven by the various ion pumps and channels, while also used to generate contractile activity of the sarcomeres. Therefore, disrupting the balance of ongoing energy generation and utilization would result in profound abnormalities in cardiac function. The ATP depletion induced by some SMKIs would activate AMPK, which along with GSK3s inhibits protein synthesis, thereby conserving energy stores. And the negative correlation between the caspase-3/7 activation and the ATP level most likely corresponded to the cellular ATP depletion with the progress of apoptosis after the 24 h of treatment. This kind of time-dependent dynamic observed for the parameter correlation can be integrated with mechanistic models to generate testable predictions in the future. Capturing the peak effect of the test compounds would enable selection of an optimum time-point for follow-up experiments.

As with any experimental model, studies with human stem cell-derived cardiomyocytes are not without limitations. While hSC-CMs have enabled a considerable breakthrough in studying cardiac biology, pharmacology and safety during the last decade, their maturation status poses a serious limitation due to potential differences in signaling pathways from those regulating the main physiological process in adult heart muscle cells. Therefore, benchmarking the target expression profiles to those in the adult human cardiomyocytes is recommended to support translation of the hSC-CM-based preclinical data. The appropriate gene expression profiles for all SMKI targets in this study was confirmed using our internal mRNA gene expression data base (data not shown). It is also important to establish the relationship between cardiotoxicity observed *in vitro* and its predicted clinical manifestation. Studying trastuzumab, sunitinib and other drugs with clinically established cardiotoxic profiles and including them as positive controls should enable setting translational standards for the *in vitro* assays in the future. An example of modeling doxorubicin-induced cardiotoxicity in hSC-CMs has shown that the *in vitro* cellular model recapitulates many of the features of this adverse drug reaction (59). These included dose-dependent increases in apoptotic and necrotic cell death, reactive oxygen species production, mitochondrial dysfunction and slowing cardiomyocyte beating rate which characterize cardiac tissue damage at the cellular level and serve as *in vitro* surrogates of the compromised clinical phenotype. Combining functional cellular monitoring with live imaging allowed us to capture changes in cellular indices for translation of the *in vitro* results into the clinic. It is expected that functionally relevant changes in these parameters at appropriate plasma concentrations would indicate a potential risk. For the clinically tested PI3K inhibitors Dactolisib, Pictilisib and Idelalisib, at least a 10-fold safety margin between the clinical exposure and the lowest *in vitro* test concentration with functionally relevant reduction in cellular indices has been determined in our hSC-CMs assay. Therefore, one would not expect to see cardiotoxicity before the free plasma concentration increases 10-fold above the expected free C_max_. Clinical trials with these drugs did not report cases of cardiac adverse events. However, the FDA adverse reaction reports from a general population showed that Idelalisib was associated with cases of atrial fibrillation, acute myocardial infarction and congestive heart failure, thus highlighting the need to further assess the cardiovascular risk of Idelalisib in postmarketing surveillance trials (60).

The real risk of cardiotoxicity is not known until the drugs become approved and used in a broader population. New tools such as hSC-CMs derived from patients will allow investigators to directly explore the mechanisms of kinase inhibitor-induced cardiotoxicity and potential mitigation strategies. Recent studies have demonstrated that patient-specific hiPSC-CMs can be utilized to reproduce the cardiotoxic effect of doxorubicin (61) and tyrosine kinase inhibitors *in vitro* (62). Furthermore, lack of significant cardiovascular co-morbidities in patients in early clinical trials (63) makes it difficult to predict true rates of cardiotoxicity for the reverse translation. The predictive value of new hSC-CMs preclinical models can potentially be determined through patient monitoring and the use of the real-world evidence in the future (64).

In summary, the present study evaluates an integrative approach to study the SMKI cardiotoxicity using hSC-CMs and reveals the main challenges of pre-clinical findings that require follow-up investigations and impact drug development programs. We foresee the introduction of new technologies to aid in discovery and to develop early safety methods for regulatory strategies translating biomedical science for novel SMKIs therapeutics into the clinic.

## Data Availability Statement

The original contributions presented in the study are included in the article/Supplementary Material, further inquiries can be directed to the corresponding author.

## Author Contributions

SK conceived the study approach and planning. RZ performed and reviewing experiments, data analysis, and contributed to writing. FH conducted the data analysis. LP did the data evaluation and writing and reviewing of the manuscript. All authors contributed to the article and approved the submitted version.

## Conflict of Interest

All authors were employed by F. Hoffmann - La Roche.
