# Supplementary figures and images for: Cardiac Safety of Kinase Inhibitors – Improving Understanding and Prediction of Liabilities in Drug Discovery Using Human Stem Cell-Derived Models
Source: Front Cardiovasc Med. 2021 Jun 16;8:639824. doi: 10.3389/fcvm.2021.639824 (PMC8242589; doi:10.3389/fcvm.2021.639824)

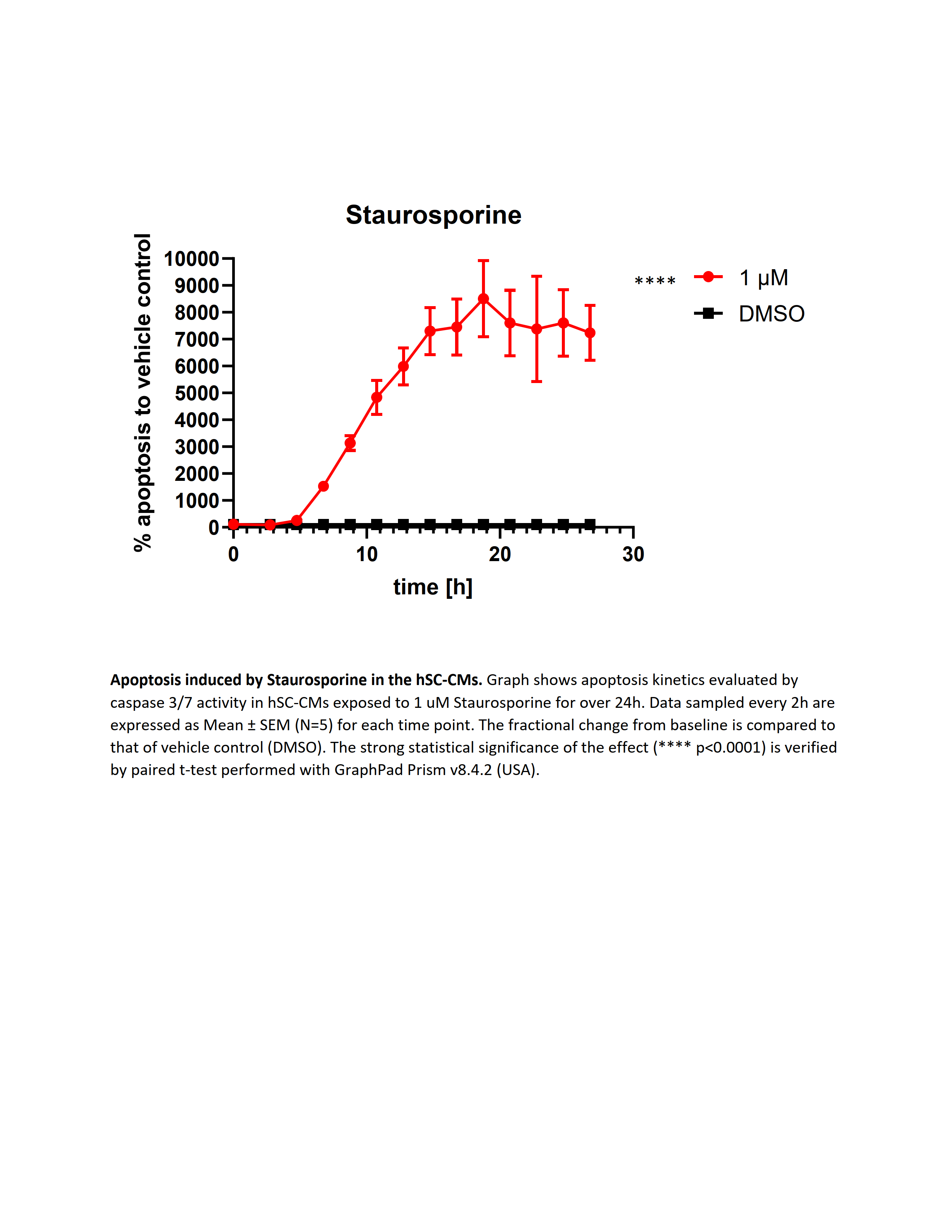

Supplement: Supplementary file 2 [file Image_1.TIF]
